# Salmonella Urinary Tract Infection and Bacteremia Following Non-Typhoidal Salmonella Gastroenteritis: An Unusual Presentation

**DOI:** 10.7759/cureus.12194

**Published:** 2020-12-20

**Authors:** Areeba Altaf, Numra Tunio, Sameer Tunio, Meer R Zafar, Numra Bajwa

**Affiliations:** 1 Internal Medicine, University at Buffalo/Catholic Health System, Buffalo, USA; 2 Internal Medicine, Ziauddin University, Karachi, PAK; 3 Internal Medicine, Aga Khan University Hospital, Karachi, PAK; 4 Internal Medicine, Sisters of Charity Hospital, Buffalo, USA; 5 Internal Medicine, Jacobs School of Medicine and Biomedical Sciences, Buffalo, USA

**Keywords:** salmonella infection, gastroenteritis, urinary tract infection, bacteremia, non-typhoidal salmonella (nts)

## Abstract

*Salmonella* is primarily known to affect the gastrointestinal tract but can rarely cause infections at uncommon sites, such as the urinary tract. It is known that *Salmonella* can infect the urinary tract directly by blood, fecal contamination of urethra, urolithiasis, or secondary intraluminal ascending infection.

Our patient is a 59-year-old female with a past medical history of nephrolithiasis and multiple urinary tract infections (UTI) who presented with altered mental status and sepsis complicated by *Salmonella* bacteremia and UTI. Urine and blood cultures revealed *Salmonella* species > 100,000 colony-forming units per milliliter (CFU/mL) and non-typhoidal *Salmonella*, respectively. During the course of her hospital admission, the patient was treated with multiple antibiotics.

On further review, it was noted that the patient had presented to the emergency room (ER) about four months earlier with abdominal pain and watery diarrhea with a stool culture being positive for non-typhoidal *Salmonella*.

Gastroenteritis, sepsis, and enteric fever are normally known with *Salmonella enterica* *serotype Typhi* (*S. Typhi*). Less common extraintestinal diseases like UTI are due to non-typhoidal *Salmonella*. The most frequent pathogenesis of *Salmonella* UTI is probably hematogenous. UTI caused by non-typhoidal *Salmonella* is usually associated with structural abnormalities of the urinary tract. In our case, the patient had non-typhoidal *Salmonella *gastroenteritis followed by non-typhoidal *Salmonella *bacteremia and UTI.

## Introduction

*Salmonella* is primarily known to affect the gastrointestinal tract but can rarely cause infections at uncommon sites like the urinary tract [1]. Non-typhoidal *Salmonella* (NTS) urinary tract infections were found to represent only 0.63% of all *Salmonella* infections in a large retrospective review [2]. It is known that *Salmonella* can infect the urinary tract directly by blood, fecal contamination of urethra, urolithiasis, or secondary intraluminal ascending infection.

Structural abnormality of the urinary tract, immunodeficiency, and chronic illness are predisposing factors for NTS urinary tract infections [3-4]. We present a case of altered mental status, nephrolithiasis, and sepsis complicated by *Salmonella* bacteremia and *Salmonella* urinary tract infection.

## Case presentation

A 59-year-old female with a past medical history of fibromyalgia, chronic obstructive pulmonary disease (COPD), allergic rhinitis, nephrolithiasis, and multiple urinary tract infections presented with altered mental status. On examination, she was febrile, tachycardic, and lethargic but arousable. No other significant examination findings, besides left flank pain and suprapubic tenderness, were noted. Her laboratory studies revealed abnormal liver enzymes, elevated creatinine and lactic acid, hypokalemia, high anion gap, and leukocytosis. Urinalysis was significant for infection.

**Figure 1:**
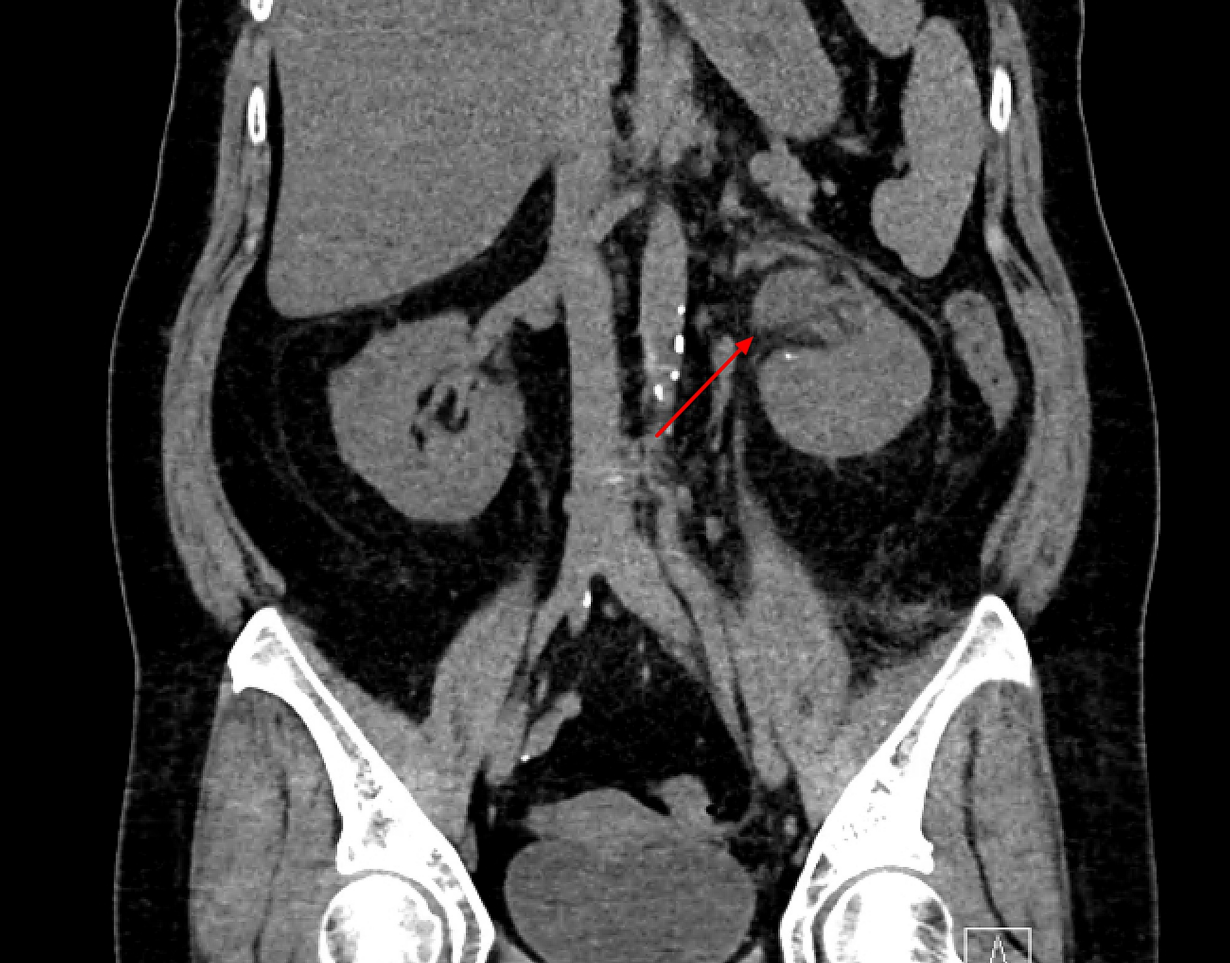
Left renal edema and hydroureteronephrosis

**Figure 2:**
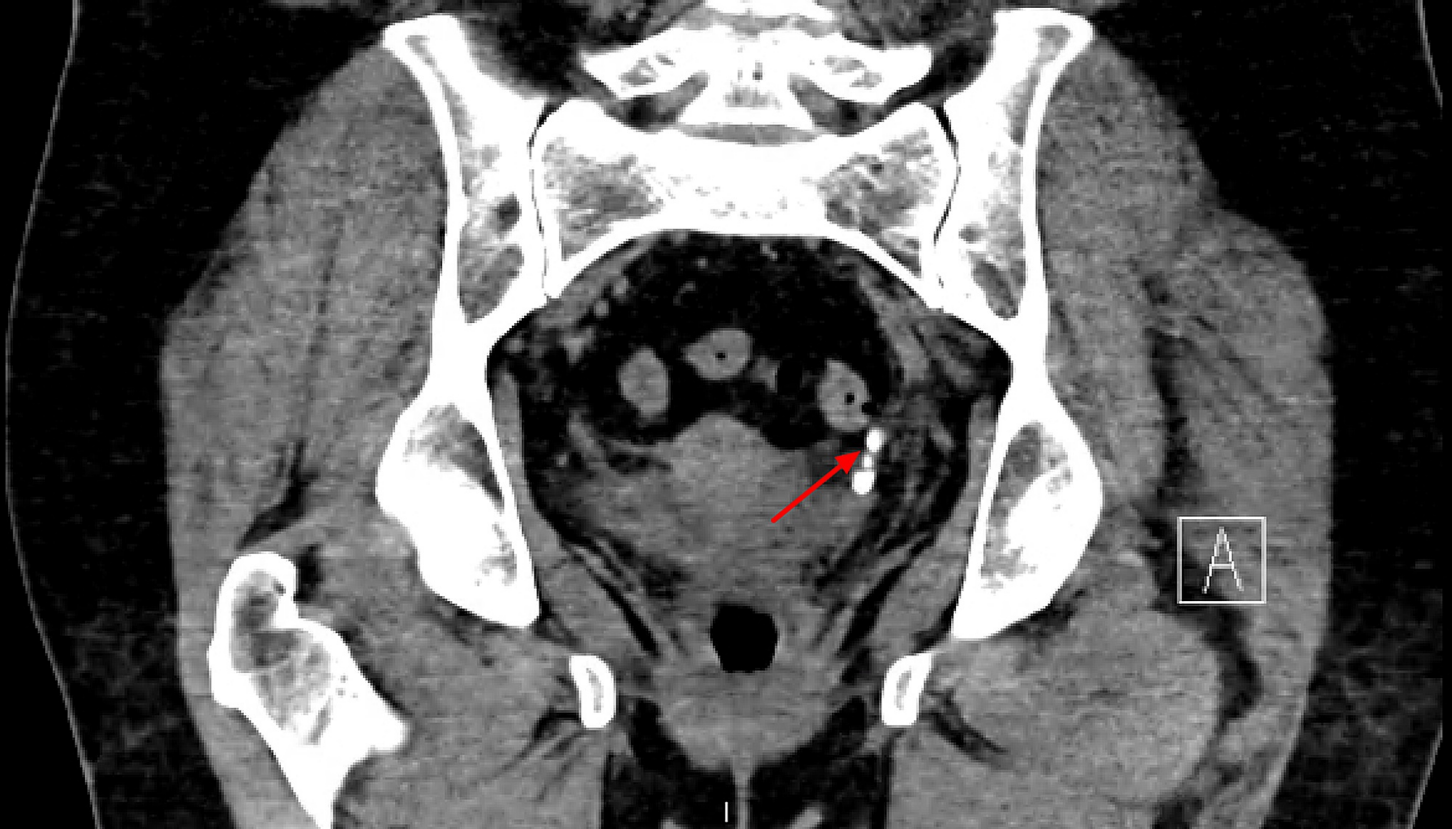
Acute obstructing calculi in the distal left ureter

Computed tomography (CT) of the abdomen/pelvis revealed acute obstructing calculi in the distal left ureter, left renal edema, and hydroureteronephrosis (Figures 1-2).

Urology was consulted, and the patient underwent left ureteroscopy with a J-stent placement. Urine and blood cultures revealed *Salmonella* species > 100,000 colony-forming units per milliliter (CFU/mL) and NTS, respectively. The patient was treated with multiple antibiotics during her hospitalization, initially started on ceftriaxone, 1 g IV daily, transitioned to ampicillin, 2 g IV q four hrs, and then eventually to levofloxacin, 750 mg PO daily, due to persistent fever and leukocytosis. Her symptoms significantly improved on levofloxacin which was also continued on discharge for a further two weeks. On further review, it was known that patient had presented to the emergency room (ER) about four months earlier with abdominal pain and watery diarrhea with a stool culture being positive for NTS. Repeat laboratory studies, urinalysis, and urine culture at the clinic one month after hospital discharge were unremarkable with the patient being completely stable with no new symptoms.

## Discussion

Gastroenteritis, sepsis, and enteric fever are normally known with *S. Typhi.* Less common extraintestinal diseases, such as urinary tract infections, are due to NTS [5]. Predisposing factors for NTS UTI include chronic diseases like diabetes, immune deficiency, or genitourinary tract abnormalities, including nephrolithiasis, chronic pyelonephritis, urethrorectal, or retrovesical fistula. It should not be ignored that NTS UTI can also occur in the healthy immunocompetent population [6].

The most frequent pathogenesis of a Salmonella upper urinary tract infection is probably hematogenous. *Salmonella* enters the body through ingestion and colonizes the ileum and colon. When *Salmonella* invades the bloodstream, it can seed distant target organs, such as the kidneys, and cause pyelonephritis. In our case, the patient had NTS gastroenteritis, followed by NTS bacteremia and UTI. Abuhasna et al. reported a similar case in which the patient had NTS UTI and was found to have blood cultures positive for NTS, making hematogenous cause a significant reason for NTS UTI [7]. *Salmonella* UTI has symptoms similar to other gram-negative UTIs, ranging from asymptomatic bacteriuria to renal abscess [8]. The reported duration of antibiotic therapy in a *Salmonella* urinary tract infection ranges from two (for mild infections) to over six weeks [9].

## Conclusions

NTS infections often present with self-limiting gastroenteritis. Urinary tract infections caused by NTS are usually associated with structural abnormalities of the urinary tract and should prompt the physician to evaluate further. Nephrocalcinosis and nephrolithiasis are the major risk factors.
